# Epstein-Barr Virus DNA Enhances Diptericin Expression and Increases Hemocyte Numbers in *Drosophila melanogaster* via the Immune Deficiency Pathway

**DOI:** 10.3389/fmicb.2018.01268

**Published:** 2018-06-11

**Authors:** Nour Sherri, Noor Salloum, Carine Mouawad, Nathaline Haidar-Ahmad, Margret Shirinian, Elias A. Rahal

**Affiliations:** Department of Experimental Pathology, Microbiology, and Immunology, American University of Beirut, Beirut, Lebanon

**Keywords:** Epstein-Barr virus (EBV), human herpesvirus 4, *Drosophila melanogaster*, diptericin, immune deficiency (IMD) pathway, tumor necrosis factor-α (TNF-α), deoxyribonucleic acid (DNA), proinflammatory

## Abstract

Infection with the Epstein-Barr virus (EBV) is associated with several malignancies and autoimmune diseases in humans. The following EBV infection and establishment of latency, recurrences frequently occur resulting in potential viral DNA shedding, which may then trigger the activation of immune pathways. We have previously demonstrated that levels of the pro-inflammatory cytokine IL-17, which is associated with several autoimmune diseases, are increased in response to EBV DNA injection in mice. Whether other pro-inflammatory pathways are induced in EBV DNA pathobiology remains to be investigated. The complexity of mammalian immune systems presents a challenge to studying differential activities of their intricate immune pathways in response to a particular immune stimulus. In this study, we used *Drosophila melanogaster* to identify innate humoral and cellular immune pathways that are activated in response to EBV DNA. Injection of wild-type adult flies with EBV DNA induced the immune deficiency (IMD) pathway resulting in enhanced expression of the antimicrobial peptide diptericin. Furthermore, EBV DNA increased the number of hemocytes in flies. Conditional silencing of the IMD pathway decreased diptericin expression in addition to curbing of hemocyte proliferation in response to challenge with EBV DNA. Comparatively, upon injecting mice with EBV DNA, we detected enhanced expression of tumor necrosis factor-α (TNFα); this enhancement is rather comparable to IMD pathway activation in flies. This study hence indicates that *D. melanogaster* could possibly be utilized to identify immune mediators that may also play a role in the response to EBV DNA in higher systems.

## Introduction

The Epstein-Bar virus (EBV) is a human pathogen that belongs to the herpes family of viruses. Like other herpes viruses, EBV establishes latency in the host after primary infection with potential future reactivation of viral replication. In addition to causing infectious mononucleosis, this virus is associated with several types of malignancies such as Burkitt and Hodgkin lymphomas, nasopharyngeal carcinoma, and post-transplant lymphoproliferative disorders (PTLD). Moreover, this virus is associated with multiple autoimmune diseases including systemic lupus erythematosus (SLE), rheumatoid arthritis (RA), and multiple sclerosis (MS) (Lünemann et al., 2007; Draborg et al., 2013). SLE patients were demonstrated to have high EBV viral loads in their peripheral blood mononuclear cells (PBMCs) (Gross et al., 2005; Yu et al., 2005). Similar observations were seen in RA patients whereby high EBV viral loads were detected in the blood, synovial fluid cells, and synovial membranes (Blaschke et al., 2000). Furthermore, various studies suggest that the risk of developing MS increases in EBV-infected individuals (Ascherio et al., 2001; Delorenze et al., 2006). A number of underlying mechanisms that link EBV to autoimmunity have been proposed; these have ranged from molecular mimicry to sequestered antigen release (reviewed in Ascherio and Munger, 2015). Nevertheless, despite these various proposed mechanisms, evidence that conclusively indicates one to mediate autoimmunity by EBV remains lacking; this likely indicates that multiple mechanisms are involved.

We have previously reported that injecting mice with EBV DNA triggers the expression of IL-17, a proinflammatory cytokine associated with autoimmune processes (Rahal et al., 2015). We also observed enhanced levels of other proinflammatory cytokines such as IL-23 and IFNγ. Others have reported that EBV DNA enhances the production of IL-8 from primary human monocytes and IFN-α from human plasmacytoid dendritic cell (Fiola et al., 2010). Owing to the fact that the latency established by EBV in the host is life-long and may result in frequent reactivations and consequent shedding of viral DNA, the proinflammatory pathways triggered by this DNA may contribute to autoimmune processes resulting in triggering or exacerbation of disease. The complexity of mammalian immune systems presents a challenge to comprehensively identify the various immune pathways triggered by EBV DNA and that may serve as potential therapeutic targets in autoimmune diseases. Hence, we attempted to establish *Drosophila melanogaster* as a relatively less complex system to detect innate immune pathways that are activated in response to EBV DNA. Furthermore, to the best of our knowledge, this is the first study examining the effect of viral DNA itself, rather than an infection, on the immune system in flies. The fly immune system is largely devoid of adaptable immune components and hence it solely relies on innate immune responses. Innate immunity in flies involves humoral as well as cellular responses. The humoral immune response involves three major pathways: the Toll, the immune deficiency (IMD), and the Janus kinase/signal transducers and activators of transcription (JAK-STAT) pathways. The production of about twenty antimicrobial peptides (AMPs) is under the control of the Toll and IMD pathways (Imler and Bulet, 2005). The expression of most of these AMPs such as attacin and cecropin may be triggered by either pathway; on the other hand, the production of drosomycin is primarily reliant on the Toll pathway while that of drosocin and diptericin is dependent on the IMD pathway. Activation of the JAK-STAT pathway has also been shown to respond to immunostimulatory challenges by triggering the expression of the stress-inducible humoral factor Turandot (TotA) (Agaisse et al., 2003). As for the cellular innate immune arm in flies it is reliant on defensive activities performed by the hemocyte compartment which 95% consists of plasmatocytes (Buchon et al., 2014). We report here that injecting EBV DNA into adult flies results in triggering the IMD pathway and in the concomitant enhancement of plasmatocyte proliferation.

## Materials and Methods

### Flies

Flies were raised and crossed at 25°C; standard *Drosophila* husbandry procedures were followed. W1118 wild-type flies (Bloomington Drosophila Stock Center #3605, Bloomington, IN, United States), UAS-STAT92e (Ghiglione et al., 2002), UAS-Relish ((Ayyar et al., 2007), and UAS-Toll10b (Maxton-Kuchenmeister et al., 1999) flies were employed. The IMD-RNAi line (Vienna Stock center #9253) was obtained from the Vienna Drosophila Stock Center (Vienna, Austria). In addition, the hemocyte driver Cg25C-GAL4 (Bloomington stock center #7011) was used.

Overexpression of Relish, Toll10b, and STAT92e was attained using the following crosses, respectively: CG25C-Gal4>UAS-Relish, CG25C-Gal4>UAS-Toll10b, and CG25C-Gal4>UAS-STAT92e. Conditional silencing of IMD was performed using the following cross: CG25C-Gal4>UAS-IMD RNAi.

### Fly Treatments

EBV DNA was obtained from Advanced Biotechnologies (Columbia, MD, United States) while bacterial DNA was prepared from an isolate of *Staphylococcus epidermidis* by phenol precipitation followed by extraction with ethanol.

To assess the effect of EBV DNA on the expression of drosomycin, diptericin, and TotA, 1-day-old flies were injected with various copy numbers of EBV DNA. As non-viral DNA control, fly groups received bacterial DNA in amounts equivalent to the weight of EBV DNA copy numbers administered. As negative controls, some fly groups received no injections while others received sterile water, the DNA solvent. Groups of 1-day-old flies that overexpressed Toll10b, STAT92e, and Relish were included as positive controls for Toll, JAK-STAT, and IMD pathway activation as well. To examine the effect of EBV DNA on older flies, 1-week-old flies were injected with EBV DNA or with sterile water. All injections were administered into the thorax of CO_2_-anesthetized flies using a Nano-injector (World Precision Instruments, Sarasota, FL, United States) and glass capillary needles. A total volume of 55.2 nl was injected into each fly.

The copy numbers of EBV DNA injected into flies were extrapolated from our previous studies in mice (Rahal et al., 2015). The following formula was used to calculate *Drosophila* equivalent copy numbers and was modified from the Food and Drug Administration (FDA) recommended formula for dose conversions (14):

$$\begin{array}{c} \mathrm{Drosophila}\;\mathrm{equivalent}\;\mathrm{dose}\;=\;\mathrm{Mouse}\;\mathrm{dose}\times \frac{\mathrm{Mouse}\;\mathrm{Km}}{\mathrm{Drosophila}\;\mathrm{Km}} \end{array}$$

Where

$$\begin{array}{c} \mathrm{Km}\;=\;\frac{\mathrm{Weight}\;(\mathrm{kg})}{\mathrm{Body}\;\mathrm{surface}\;\mathrm{area}\;(m^{2})} \end{array}$$

The body surface area of *D. melanogaster* was calculated using Mosteller’s formula (Mosteller, 1987).

### Gene Expression Studies in Flies

Ten flies were collected per group per time point for gene expression studies. RNA extraction was subsequently performed using TRIzol (Sigma*-*Aldrich, St. Louis, MO, United States) according to the manufacturer’s specifications. Then, cDNA synthesis was carried out using the QuantiTect^®^ Reverse Transcription Kit (QIAGEN, Hilden, Germany). Real-time reverse transcriptase PCR was then performed to analyze relative gene expression levels. Primers used for this purpose were obtained from Thermo Scientific (Ulm, Germany). Previously published primers (**Table 1**) were used to analyze the expression of the drosomycin (Tanji et al., 2007), diptericin (Avadhanula et al., 2009), and TotA (Kallio et al., 2010) genes. Primers used to assess the expression of the IMD gene were selected using the NCBI primer designing tool. Transcription of the house keeping gene RPL32, used as an internal control, was detected also using previously published primers (Hedges and Johnson, 2008). Real-time PCR reactions each consisted of 10 μl and contained 5 μl of SYBR green, 150 pmoles of the forward primer, 150 pmoles of the reverse primer, and 150 ng of cDNA. Samples were analyzed in triplicates. Real time detection was performed in a BioRad CFX96 Real Time System employing a C1000 Thermal Cycler (Munich, Germany). A PCR initial activation step of 95°C for 5 min was used followed by 40 cycles of 95°C for 15 and 30 s at the annealing temperature for each primer as published. Relative gene expression normalized to the water-injected group was calculated using the ΔΔCq method. Samples were analyzed in triplicates.

**Table 1:** Primer sequences.

Primer	Sequences	Reference
Drosomycin	F: 5′-TACTTGTTCGCCCTCTTCG-3′ R: 5′-GTATCTTCCGGACAGGCAGT-3′	Tanji et al., 2007
Diptericin	F: 5′-AAGTGGGAAGCACCTACACCTACA-3′ R: 5′-GTATCTTCCGGACAGGCAGT-3′	Avadhanula et al., 2009
TotA	F: 5′-CCCAGTTTGACCCCTGAG-3′ R: 5′-GCCCTTCACACCTGGAGA-3′	Kallio et al., 2010
IMD	F: 5′-TCAGCGACCCAAACTACAATTC-3′ R: 5′-TTGTCTGGACGTTACTGAGAGT-3′	(Using NCBI primer designing tool)
RPL32	F: 5′-GACGCTTCAAGGGACAGTATCTG-3′ R: 5′-AGGGCCACAGCATGGGTCTGT-3′	Hedges and Johnson, 2008

### Hemocyte Count

Flies were examined for hemocyte counts as previously described (Ghosh et al., 2015). For this purpose, three female flies per time point from each assessed group were anesthetized with CO2 and then their wings were excised. The flies were then placed in phosphate buffered saline (PBS) using 10 μl per fly. A fine incision was used to expose the thorax of each fly and bleeding was allowed into the PBS for 20 s. Subsequently, hemocyte counts were determined by examining 10 μl of the bleed using a hemocytometer under a light microscope and employing a 40× magnification. Analysis was performed in triplicates for each time point.

### Analysis of TNFα Expression in Mice

Experimental procedures conducted on mice were approved by the Institutional Animal Care and Use Committee (IACUC) at the American University of Beirut (AUB). BALB/c mice, 4-6 weeks of age, were obtained from the Animal Care Facility at AUB. Mice were intraperitoneally injected with 144 × 10^3^ copies of EBV DNA, 22.3 pg of *S. epidermidis* DNA (equivalent to the weight of 144 × 10^3^ EBV DNA copies) or sterile water. Each injection consisted of 100 μl. Three mice per treatment were sacrificed on day 6 post-injection, their spleens were collected, pooled and then subjected to RNA extraction using TRIzol. The RNA was used for cDNA synthesis using the QuantiTect^®^ Reverse Transcription Kit. Real-time reverse transcriptase PCR was then performed to detect relative gene expression levels of TNFα using previously published primers (Peinnequin et al., 2004). Previously published primers (Pindado et al., 2007) were also employed to detect the expression of β-actin, used as an internal control. Real-time PCR reactions were performed and relative gene expression normalized to the water-injected mice was calculated as described for fly gene expression studies described above. Analysis was conducted in triplicates.

### Statistical Analysis

To analyze statistical significance unpaired *t*-tests were used to compare means and were performed using Graphpad; *p*-values less than 0.05 were considered statistically significant.

## Results

### EBV DNA Enhances the Expression of Diptericin in Flies

**FIGURE 1:**
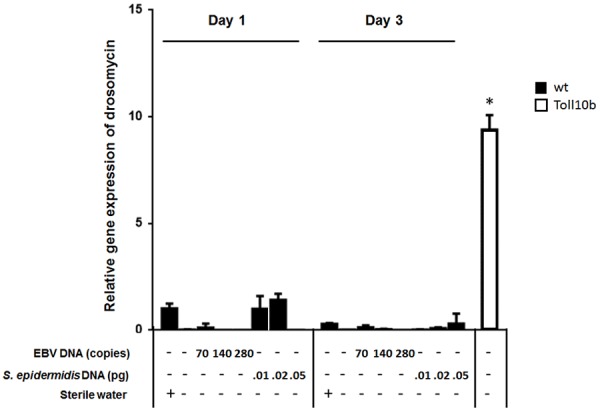
Drosomycin relative gene expression in flies injected with EBV DNA on days 1 and 3 post-injection. Expression was assessed in W1118 wild-type (wt) flies that received no injection and wt flies injected with sterile water, EBV DNA (70, 140, or 280 copies) or *S. epidermidis* DNA (0.01, 0.02, or 0.05 pg) on days 1 and 3 post-injection. Flies overexpressing Toll10b were included as a positive control for drosomycin expression. Data is normalized to expression in water-injected flies on day 1 post-injection. ^∗^Indicates *p* < 0.05.

**FIGURE 2:**
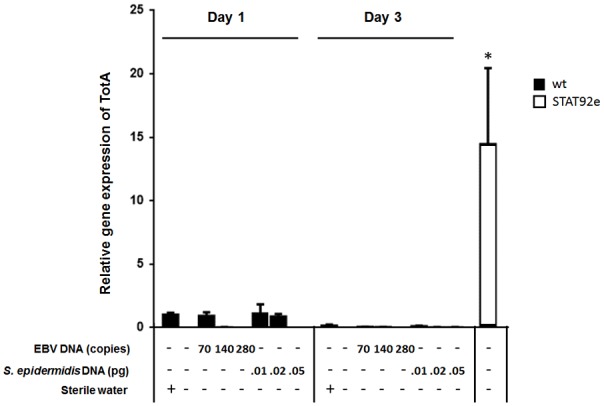
TotA relative gene expression in flies injected with EBV DNA on days 1 and 3 post-injection. Expression was assessed in W1118 wild-type flies (wt) that received no injection and wt flies injected with sterile water, EBV DNA (70, 140, or 280 copies) or *S. epidermidis* DNA (0.01, 0.02, or 0.05 pg) on days 1 and 3 post-injection. Flies overexpressing STAT92e were included as a positive control for TotA expression. Data is normalized to expression in water-injected flies on day 1 post-injection. ^∗^Indicates *p* < 0.05.

**FIGURE 3:**
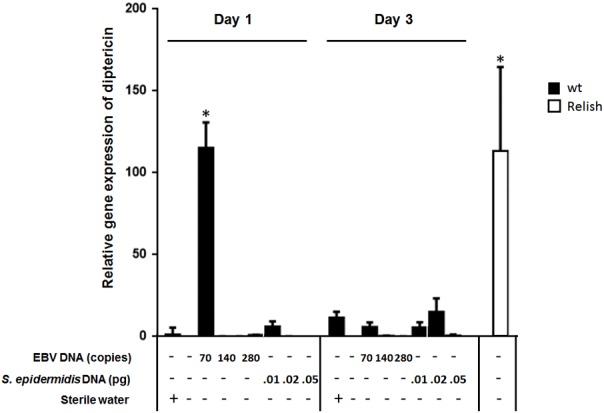
Diptericin relative gene expression in flies injected with EBV DNA on days 1 and 3 post-injection. Expression was assessed in W1118 wild-type (wt) flies that received no injection and wt flies injected with sterile water, EBV DNA (70, 140, or 280 copies) or *S. epidermidis* DNA (0.0.1, 0.02, or 0.05 pg) on days 1 and 3 post-injection. Flies overexpressing Relish were included as positive control for diptericin expression. Data is normalized to expression in water-injected flies on day 1 post-injection. ^∗^Indicates *p* < 0.05.

To assess the effect of EBV DNA on the humoral arm of innate immunity in *D. melanogaster*, flies were intrathoracically injected with 70, 140, or 280 copies of EBV DNA. Flies were then analyzed for the expression of drosomycin, diptericin, and TotA as molecular indicators of activation of the Toll, IMD, and JAK-STAT pathways, respectively. Whereas we did not observe an increase in the expression of drosomycin (**Figure 1**) or TotA (**Figure 2**) upon EBV DNA treatment, we detected a 115-fold increase (*p* = 0.0002) in the transcriptional levels of diptericin (**Figure 3**) in the group injected with 70 copies of EBV DNA on day 1 post-injection. This level then decreased to 5.62 on day 3. No notable changes were observed upon injecting flies with 140 or 280 copies of EBV DNA.

To examine whether the observed effects are specific to EBV DNA itself, flies were injected with an amount of *S. epidermidis* DNA equivalent to the weight of the EBV DNA copy numbers employed. Treatment with this non-viral DNA control did not notably alter the expression of the humoral immunity markers assessed.

**FIGURE 4:**
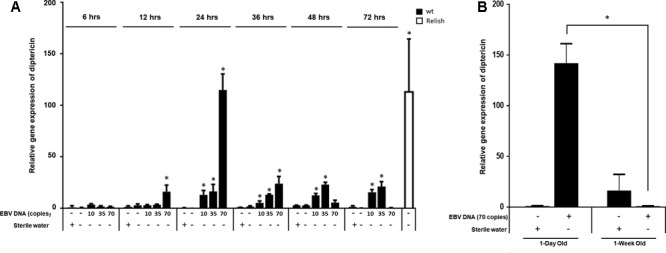
Diptericin relative gene expression in flies injected with EBV DNA at 6, 12, 24, 36, 48, and 72 h post-injection. **(A)** Expression was assessed in W1118 wild-type (wt) flies that received no injection and wt flies injected with sterile water, EBV DNA (10, 35, or 70 copies) or *S. epidermidis* DNA (0.0.1, 0.02, or 0.05 pg) at multiple time points post-injection. Flies overexpressing Relish were included as positive control for diptericin expression. Data is normalized to expression in water-injected flies at 6 h post-injection. ^∗^Indicates *p* < 0.05. **(B)** Diptericin relative gene expression was assessed in 1-day-old and 1-week-old W1118 wild-type (wt) flies after 24 h of receiving an injection of 70 copies of EBV DNA. Flies that received injections of sterile water were included as controls. ^∗^Indicates *p* < 0.05.

We then examined the expression of diptericin at 6, 12, 24, 48, 36, and 72 h after injection (**Figure 4A**) and included lower copy numbers of EBV DNA into the analysis. Upon injecting flies with 10 copies of EBV DNA, diptericin levels began to significantly increase after 24 h of injection and continued to elevate reaching a highest detected increase of 16-folds (*p* = 0.001) 72 h after injection. Flies injected with 35 copies of EBV DNA also demonstrated significantly increasing levels of diptericin expression after 24 h of injection with levels peaking at 48 h after injection (23-folds, *p* = 0.0001) then decreasing afterwards. Injection of 70 copies of EBV DNA resulted in a significant increase in the relative gene expression starting after 12 h of injection, peaking at 24 h and decreasing from then onwards. Hence, a time-dependent dose response is seen in the enhancement of diptericin expression by EBV DNA treatment. Injection with 140 or 280 copies did not result in any increase in the levels of diptericin expression indicating that EBV DNA may have a biphasic hormetic effect on diptericin expression in flies whereby low levels are stimulatory but high levels are inhibitory. To examine the effect of EBV DNA in older flies, 1-week-old wild-type flies were injected with 70 copies of EBV DNA; diptericin expression levels were then determined in these flies after 24 h of receiving the injection. Diptericin expression levels were reduced by about 140-folds in the 1-week-old flies upon injection with EBV DNA compared to similarly treated 1-day old flies (**Figure 4B**).

### Silencing IMD Abrogates EBV DNA-Triggered Expression of Diptericin

**FIGURE 5:**
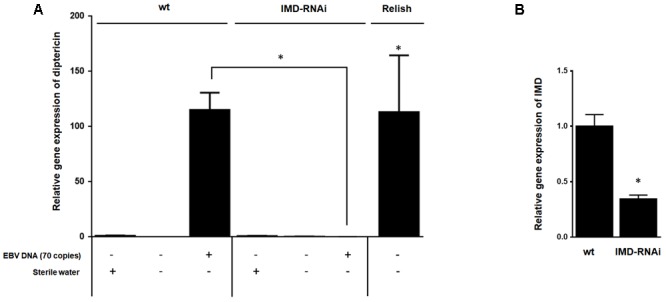
Effect of IMD silencing on diptericin relative gene expression in flies injected with EBV DNA. **(A)** IMD relative gene expression in W1118 wild-type (wt) and IMD knock-down flies. **(B)** Diptericin expression was assessed in W1118 wild-type (wt) and IMD knock-down flies that received no injection, were injected with sterile water or with EBV DNA (70 copies). Expression was assessed on day 1 post-injection. Flies overexpressing Relish were included as a positive control for diptericin expression. ^∗^Indicates *p* < 0.05.

To establish that enhanced expression of diptericin in response to EBV DNA is the result of IMD pathway activation rather than an alternate pathway, we knocked down the expression of the IMD mediator, a key component of this pathway. Flies were then injected with 70 copies of EBV DNA and analyzed for expression of diptericin after 24 h of injection (**Figure 5A**). In contrast to wild-type flies, knocking down IMD resulted in inhibition of diptericin expression in response to EBV DNA (*p* = 0.009). Assessing the efficiency of silencing IMD demonstrated a substantial decrease in the mRNA levels (**Figure 5B**).

### EBV DNA Elevates the Number of Hemocytes in Fly Bleed

**FIGURE 6:**
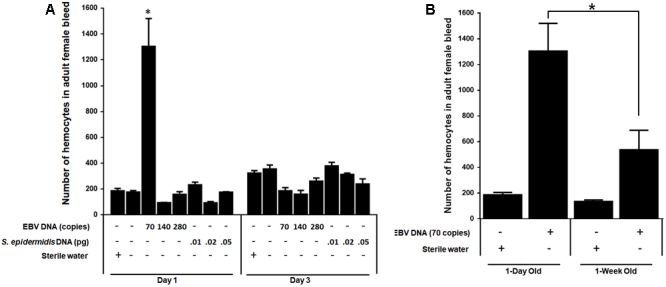
Number of hemocytes in flies injected with EBV DNA. **(A)** Hemocytes in adult female fly bleed were enumerated in W1118 wild-type (wt) flies that received no injection and wt flies injected with sterile water, EBV DNA (70, 140, or 280 copies) or *S. epidermidis* DNA (0.0.1, 0.02, or 0.05 pg) on days 1 and 3 post-injection. ^∗^Indicates *p* < 0.05. **(B)** Hemocytes were enumerated in 1-day-old and 1-week-old W1118 wild-type (wt) flies after 24 h of receiving an injection of 70 copies of EBV DNA. Flies that received injections of sterile water were included as controls. ^∗^Indicates *p* < 0.05.

We examined the effect of EBV DNA on the cellular arm of fly immunity by injecting adult female flies with 70, 140, or 280 copies of EBV DNA and then assessing the number of hemocytes in the fly bleed on days 1 and 3 post-injection. Treatments with *S. epidermidis* DNA were included as a non-viral DNA control. We observed a sevenfold increase (*p* = 0.0009) in the number of circulating hemocytes in flies administered 70 copies of EBV DNA after 1 day of injection. This number had notably dwindled back to normal levels on day 3 after injection in this group. As for flies injected with 140 or 280 copies of EBV DNA or with *S. epidermidis* DNA no notable elevations in circulating hemocyte numbers were detected (**Figure 6A**). To examine the effect of EBV DNA in older flies, 1-week-old wild-type flies were injected with 70 copies of EBV DNA; hemocytes were then enumerated after 24 h of receiving the injection. The number of hemocytes in the bleed of 1-week-old flies upon injection with EBV DNA was about 30% lower than that in 1-day-old flies that were similarly treated (**Figure 6B**).

### The IMD Pathway Plays a Role in the EBV DNA-Triggered Elevation of Hemocyte Levels

**FIGURE 7:**
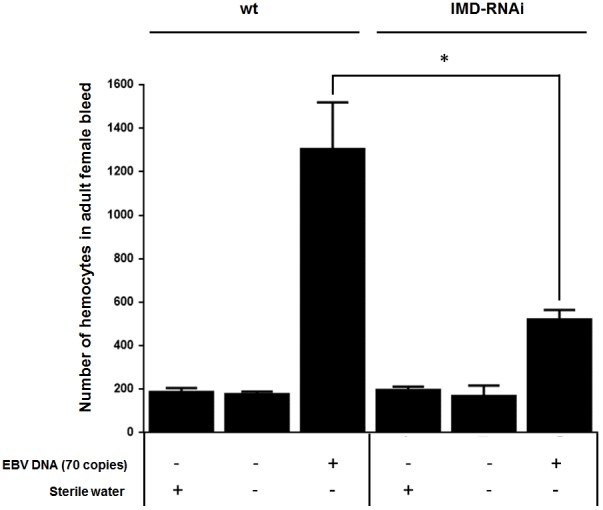
Number of hemocytes in IMD-silenced flies injected with EBV DNA. Number of hemocytes in adult female fly bleed in W1118 wild-type (wt) and IMD knock-down flies that received no injection, were injected with sterile water or with EBV DNA (70 copies). Cells were enumerated on day 1 post-injection. ^∗^Indicates *p* < 0.05.

Both circulating hemocyte numbers and diptericin levels were elevated in response to 70 copies of EBV DNA after 24 h of injection but not by 140 or 280 copies of DNA or at later time points. Thus, circulating hemocyte numbers were affected by EBV DNA in a manner that highly mirrored the observed responses in diptericin expression within EBV DNA-treated fly groups. Consequently, we analyzed whether the IMD pathway underlies this elevation in hemocytes by knocking down the IMD mediator and examining the number of hemocytes in the bleed after 24 h of injecting adult female flies with 70 copies of EBV DNA (**Figure 7**). Knocking down IMD decreased the number of hemocytes 2.5-folds (*p* = 0.0035) compared to wild-type flies upon injection with 70 copies of EBV DNA. This indicates that this pathway is a key player in the cellular responses seen upon treating flies with EBV DNA.

### EBV DNA Enhances TNFα Expression in Mice

**FIGURE 8:**
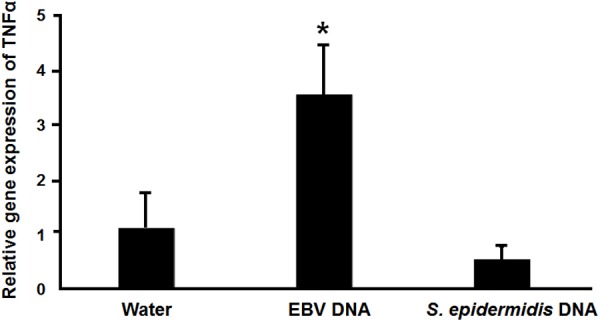
Relative gene expression of TNFα in mice injected with EBV DNA. Expression was assessed in splenic tissue from BALB/c mice that were intraperitoneally injected with water, 144 × 10^3^ copies of EBV DNA or 22.3 pg of *S. epidermidis* DNA on day 6 post-injection. ^∗^Indicates *p* < 0.05.

The IMD pathway is often compared to tumor necrosis Factor-α Receptor signaling (TNFR) in mammals (71). Hence, we examined whether EBV DNA triggers TNFα in a mammalian system by injecting mice with 144 × 10^3^ copies of EBV DNA and assessing TNFα expression in mouse spleens on day 6 post-injection (**Figure 8**). We had previously detected a peak in mouse IL-17 levels on that day after injection with 144 × 10^3^ copies of EBV DNA. Mice injected with EBV DNA showed a 3.5-fold increase in the transcription of TNFα. On the other hand, no significant changes were detected in mice injected with bacterial DNA.

## Discussion

Enhanced production of proinflammatory cytokines triggered by EBV DNA in mammalian systems has been reported by our group among others (Fiola et al., 2010; Rahal et al., 2015). This raises the possibility of involvement of other immune modulators in response to EBV DNA. We thus aimed to implement a system that permits relatively simple screening of humoral and cellular responses to viral DNA. Therefore, we used the fruit fly, *D. melanogaster*, an organism with a versatile library of genetic tools to study such responses. The fruit fly has well-conserved cellular and humoral innate immune pathways in addition to the absence of adaptive immunity; this facilitates the identification of innate pathways, the basis of proinflammatory responses that may be involved in EBV DNA-triggered immune activation in higher mammalian systems.

Upon injecting 70 copies of EBV DNA into flies, the transcriptional level of diptericin, the hallmark of IMD pathway activation, was significantly increased by day 1 post-injection. In a similar manner, hemocyte numbers were significantly elevated in flies upon treatment with EBV DNA. Both diptericin expression and hemocyte proliferation effects had dwindled by day 3 post-injection. On the other hand, injections of 140 or 280 copies of EBV DNA did not result in similar increases in neither diptericin expression levels nor in hemocyte proliferation. This may indicate that EBV DNA results in a biphasic hormetic response in flies whereby lower doses induce a possibly beneficial immunostimulatory response whereas higher doses overwhelm the system and are rather inhibitory. Various agents have been previously described to have such an effect in biological systems (Calabrese and Baldwin, 1999, 2001). The particular underlying molecular etiology of such a response remains to be elucidated. Worth noting is that an injection of 144 × 10^3^ copies of EBV DNA in mice, which we had previously reported to induce a prominent elevation of IL-17A levels and demonstrated herein to induce TNFα expression in mice, is equivalent to administration of 140 copies of EBV in flies; whereas this dose appears to be immunostimulatory in mice, a similar observation is not seen in flies. Therefore, whether higher doses of EBV DNA also have a biphasic hormetic immunomodulatory response in mice remains to be investigated. Knocking down IMD abrogated both of the humoral and cellular effects. These results suggest that cross-talk between humoral and cellular pathways may occur in response to EBV DNA injection and that IMD is a key mediator in both types of activation. The IMD pathway has been shown to induce Jun amino-terminal kinase (JNK)-dependent expression of the ligands for *Drosophila* vascular endothelial growth factor (VEGF) and platelet-derived growth factor (PDGF) receptor (PVR), PDGF- and VEGF-related factors 2 and 3 (PVF2 and PVF3) (Bond and Foley, 2009), which in turn induce hemocyte proliferation in *Drosophila* (Munier et al., 2002). Hence, the increase in diptericin expression levels and hemocyte numbers upon EBV DNA injection is likely initiated by activation of the IMD humoral immune pathway; this subsequently results in cellular responses. The observation that 70 or fewer copies of EBV DNA trigger the IMD pathway but that 140 or 280 copies do not induce such a response likely indicates that higher copy numbers exert an inhibitory effect; the particular nature of this effect was not determined by the current study. Worth noting is that the DNA within a nascent EBV virion upon release from an infected cell is linear and contains unmethylated CpG dinucleotides (Kintner and Sugden, 1981). Hence, any shed DNA in addition to that delivered into a cell upon an infection is in this form. Hence, the DNA we used in the study at hand was also linear and unmethylated.

The transcriptional levels of drosomycin and TotA, which are indicative of Toll and JAK-SAT pathway activation, respectively, were not affected by EBV DNA treatment. This may indicate that EBV DNA does not activate these pathways or that their activation occurs under other conditions than those assessed by the current study. Previous studies have indicated that unmethylated CpG DNA motifs, which are abundant in the EBV genome in a nascent viral particle (Bergbauer et al., 2010), activate innate immunity through TLR9 in mammals (Fiola et al., 2010); hence, *D. melanogaster*, as implemented for examining the immune effects of EBV DNA in the current study, may be used to assess Toll involvement in the response to EBV DNA in flies under other conditions in further studies. Worth noting is that in *D. melanogaster* endogenous accumulation of chromosomal DNA also triggers the production of diptericin (Häcker et al., 2000). Although the nature of DNA exerting this effect is not microbial, it may indicate that responses to various types of immunostimulatory DNA occur via activation of the IMD pathway in flies.

The IMD pathway in flies is considered to be comparable to signaling mediated by TNFR in mammals (Govind, 2008). We hence examined whether TNFα expression levels are affected by EBV DNA in mice. We observed elevated expression levels of this proinflammatory mediator. Hence, a congruent response was detected in mice. We have previously reported that IL-17, IL-23, and IFNγ are enhanced in mice administered EBV DNA. The detection of elevated TNFα adds to the complexity of the proinflammatory response that seems to be triggered in mammalian systems in response to EBV DNA.

## Conclusion

EBV DNA triggers the IMD pathway in flies and enhances comparable TNFα expression in mice. This rather validates the implementation of D. melanogaster to screen for responses and mediators triggered by viral DNA. Identification of sensors of viral DNA and downstream mediators via the use of the fruit fly may reveal possible therapeutic targets of pro-inflammatory responses in humans.

## Author Contributions

NSh participated in the study design, performed the experiments, and contributed to analysis and writing. NSa, CM, and NH-A assisted in experimental procedures. MS and ER designed the study, supervised the work and resultant analysis. MS and ER contributed equally to this work and should both be listed as corresponding authors.

## Conflict of Interest Statement

The authors declare that the research was conducted in the absence of any commercial or financial relationships that could be construed as a potential conflict of interest. The reviewer HZ and handling Editor declared their shared affiliation.
